# The Associations between Self-Consciousness, Depressive State and Craving to Drink among Alcohol Dependent Patients Undergoing Protracted Withdrawal

**DOI:** 10.1371/journal.pone.0071560

**Published:** 2013-08-27

**Authors:** Philippe de Timary, Mariana Cordovil de Sousa Uva, Catherine Denoël, Ludger Hebborn, Marc Derely, Martin Desseilles, Olivier Luminet

**Affiliations:** 1 Institute of Neuroscience, Université catholique de Louvain, Louvain-la-Neuve, Belgium; 2 Research Institute for Psychological Sciences, Université catholique de Louvain, Louvain-la-Neuve, Belgium; 3 The Belgian National Fund for Scientific Research (FRS-FNRS), Brussels, Belgium; 4 Depression Clinical and Research Program, Department of Psychiatry, Massachusetts General Hospital, Harvard Medical School, Boston, Massachusetts, United States of America; 5 Department of Psychology, University of Namur, Namur, Belgium; 6 Unité d'Hépatologie Intégrée Department of Adult Psychiatry and Institute of Neuroscience, Academic Hospital Saint-Luc, Université catholique de Louvain, Brussels, Belgium; 7 Psychiatry Ward, Clinique Europe St Michel, Brussels, Belgium; 8 The Alcohol Dependence Unit, Clinique La Ramée, Brussels, Belgium; Catholic University of Sacred Heart of Rome, Italy

## Abstract

**Context:**

In order to understand how certain personality traits influence the relation between depression symptoms and craving for alcohol, trait self-consciousness (trait SC) was examined during a withdrawal and detoxification program.

**Methods:**

Craving (Obsessive and Compulsive Drinking Scale), depressive state (Beck Depression Inventory) and trait SC (Revised Self-Consciousness Scale) were assessed in alcohol-dependent inpatients (DSM-IV, N = 30) both at the beginning (T1: day 1 or 2) and at the end (T2: day 14 to18) of protracted withdrawal during rehabilitation.

**Results:**

A significant decrease in craving and depressive symptoms was observed from T1 to T2, while SC scores remained stable. At both times, strong positive correlations were observed between craving and depression. Moreover, regression analyses indicated that trait SC significantly moderated the impact of depression on cravings for alcohol.

**Limitations:**

This study was performed on a relatively small sample size. Administration of medications during detoxification treatment can also be a confounding factor. Finally, craving could have been evaluated through other types of measurements.

**Conclusions:**

During protracted withdrawal, alcohol craving decreased with the same magnitude as depressive mood. Depressive symptoms were related to alcohol craving but only among patients with high trait SC scores. Our results suggest that metacognitive approaches targeting SC could decrease craving and, in turn, prevent future relapses.

## Introduction

While under the influence of alcohol, alcohol-dependent patients usually experience negative affects including depressive states [1], [2] which have been shown to decrease as a result of alcohol withdrawal treatment [3]–[6]. Similarly, “craving” or strong appetitive urge to drink alcohol, another central component of alcohol-dependence [7], decreases during withdrawal treatment [6], [8], [9]. Craving is a subjective feeling that is experienced by many addicts [7] and is strongly associated with any future relapses after withdrawal treatment [10]–[12]. Among the numerous mechanisms that may underlie craving, a recent cognitive-affective model proposed by Baker and colleagues [13], [14] suggests an interaction between drug motivation and affective symptoms. They argued that the phenomenon of craving is most often elicited by a negative emotion processing system, where drinking is used as a mean to escape negative affect. Although such negative affects like depression have never been experimentally demonstrated to be a causal agent in the motivation to drink, the associations between depression and craving for alcohol were found in a few studies. Indeed, by using self-reporting questionnaires to examine drinking motivation (or craving), several authors have found positive correlations between depressive state and craving for alcohol during withdrawal treatment [6], [15]. Furthermore, more recent research has also found relations between personality traits and craving [9], [16]. In particular, self-consciousness (SC), a trait which refers to the propensity to direct ones attention to the self has been found to be related to negative affects and alcohol-drinking [17]. As a result, Hull and his colleagues developed a theory of drinking which suggests that drinking alcohol should decrease or even inhibit the level of SC in alcohol-dependent subjects [17]. In their original study, they examined the moderating effect of SC on the relation between positive and negative life events, on the one hand, and the behavioral outcome of relapse following detoxification of alcohol-dependent subjects on the other hand. They observed that individuals scoring high on SC and who experienced events related to personal failure were more prone to alcohol relapse. They argued that this occurred because they were more sensitive to the negative self-relevant implications of such events. Conversely, when they did not meet situations where they experienced personal failure, high SC scorers were not motivated to reduce self-awareness and hence did not exhibit high relapse rates [17]. These results support the idea that alcohol consumption interferes with the encoding process of information relevant to the self, probably as an escape mechanism when this information can provide a source of self-criticism and negative affect. A recent study suggests that the interference with the encoding of self-referent information is necessary in alcohol-dependent subjects because they present with exaggerated high standards [18]. Nevertheless, a correct state of self-awareness is necessary for one to adopt appropriate behaviours in everyday situations, especially when subjects are expected to change their attitudes [19]. In alcohol-dependent subjects, the altered state of self-awareness will certainly slower the initiation of changes in addictive behaviours. For this reason, psychological interventions that aim at motivating changes in drinking behaviour will largely target an increase in self-awareness of alcohol dependent patients [20].

Despite this research, to our knowledge, trait SC has yet to be examined during the withdrawal treatment of alcohol-dependent patients. Alcohol-withdrawal is an important initial step in the treatment of alcohol-dependence and allows for the observation of, not only changes in craving and depression scores, but also how SC may moderate the relation between these factors. Moreover, since craving is a strong predictor of relapse after a treatment for alcohol-dependence [10]–[12] the moderation by SC of the relation between depression and craving might provide a better understanding of the intermediate processes that explain relapse after protracted withdrawal. In other words, relapse might be explained in high SC scorers by the elicitation of craving in situations of depression.

Thus, the present study has two primary aims: 1.) to evaluate changes in depressive symptoms and their relations with craving during a protracted withdrawal and detoxification program among alcohol-dependent patients and 2.) to test whether trait SC moderates the relation between depression and craving for the same people. In keeping with the self-awareness model of drinking [21], we hypothesized that patients with a high level of SC and with depressive symptoms will exhibit more craving for alcohol in order that drinking decreases their state level of SC.

## Methods

### Participants

The current study was accepted by the Commission d'éthique hospitalo-facultaire of the medical faculty of Université Catholique de Louvain, Avenue Hippocrate 55.14, 1200 Brussels and all patients signed an informed consent form that was approved by the ethical committee. The participants were totally free to join into the study and all patients that declined to participate were not disadvantaged by any way by not participating into the study. Forty patients meeting the DSM-IV criteria for alcohol dependence [22] were clinically evaluated and recruited by three psychiatrists (P.d.T., LH or M.D.) from a detoxification program at the 1) Unité Intégrée d'Hépatologie, Department of Adult Psychiatry at the Academic Hospital St. Luc, 2) alcohol dependence unit of Clinique La Ramée and 3) the Psychiatry ward of Clinique Europe St. Michel in Brussels (Belgium). Only 30 (75%) (19 males and 11 females) were tested at two times of protracted withdrawal during rehabilitation: at the beginning (T1: day 1 or 2) and at the end (T2: day 14 to18). The 10 drop-out patients were unable to finish the study either because they were not able to continue the testing owing to tiredness, pain or distress or because they interrupted their stay before the second testing (frequently owing to relapse). In order to encourage participation, patients were informed that their involvement in the research would help provide a better understanding of alcohol-dependence. No financial or material incentive was provided. Only those who drank alcohol on the date of application to the program or the day before were included in the study. Patients who were addicted to substances other than alcohol (and cigarettes) such as illicit drugs or benzodiazepines were excluded from the study. Indeed, these substances may also influence their affective and craving states and the withdrawal duration of these substances is different from that of alcohol [23]. Patients systematically received benzodiazepines (diazepam: usually 30 to 40 mg per day) at the beginning of the treatment to minimize alcohol withdrawal syndrome. The dosage was adapted as a function of body-mass and progressively tapered out within one or two weeks during the detoxification program. Vitamin B1 was also given to all patients to decrease the risk of developing Gayet-Wernicke syndrome. The current study met all ethical standards as stated by the ethics committees at each hospital and all patients signed an informed consent form.

### Measures

Two state-related dimensions (cravings and depression) and personality trait SC were measured via self-report questionnaires.

*The Obsessive-Compulsive Drinking Scale (OCDS)* measures cognitive aspects of alcohol craving over the last 7 days at the time of filling out the measure [24], [25]. OCDS is a self-report craving questionnaire that is comprised of a total (Tot) of 14 items, which can be sub-divided into two subscales, a 6-item ‘obsessive’ subscale (Ob) (e.g., *How much of your time when you're not drinking is occupied by ideas, thoughts, impulses, or images related to drinking?)* and an 8-item ‘compulsive’ subscale (Co) (e.g., *How much of an effort do you make to resist consumption of alcoholic beverages?*). All items are rated along 5-point *Likert* scale (0 = least, 4 = most) referring to 5 statements which express the degree of the severity of craving. Four compulsive items are related to alcohol consumption *(e.g., How many drinks do you drink each day?)*. These items corresponded to items number 7, 8, 9 and 10 of Anton's questionnaire (Anton et al., 1995, 1996). As these questions are related to alcohol consumption and alcohol consumption is prohibited during withdrawal these items represent an inaccurate index of compulsion to drink at T2. Thus they were eliminated and a modified 4-item compulsive subscore (Com) and a modified 10 item total score (Totm) was computed. The French version of the scale used for the present study has been validated by Ansseau and his colleagues [26].

*The Beck Depression Inventory (BDI)* is a self-report inventory designed to measure severity of depressive symptoms. The BDI is widely considered an accurate measure of depressive state severity in alcohol-dependent patients [27], [28]. The French translation of the second version of BDI (BDI-II) [29] used in this study has been validated [30]. BDI-II consists of 21 items measuring characteristic attitudes and symptoms of depression (e.g., *sadness, tiredness*). The items are rated along a 4-point Likert scale (0 = least, 3 = most) referring to 4 statements which express severity depression degree. The total score is the sum of all items. All questions refer to the feelings experienced by the patient in ‘the past week, including today’ and is, therefore, a state measure of depression.

*The Revised Self-Consciousness Scale (RSCS)* assesses individual levels of trait SC [31]. Self-consciousness (SC) is a personality trait characterized by a tendency to think and to direct attention towards the self. This involves chronic self-focused attention in which a person focuses on his own thoughts, feelings, behaviours or appearance; reflections, fantasies, or daydreams about himself; or makes decisions or plans involving himself [32]. This scale includes 22 items rated on a 4-point scale ranging from “Extremely uncharacteristic” to “Extremely characteristic” under three main separate facets of SC. The first facet is Private Self-Consciousness. This facet reflects different concerns about self-thoughts, internal feelings and focuses on personal and hidden reflections dealing with the self (e.g., *I generally pay attention to my inner feelings*). The second facet, Public Self-Consciousness, reflects a persons' awareness of another individuals' perspective. Items in this subscale also assess the extent to which an individual cares about his appearance in front of others (e.g., *I care a lot about how I present myself to others*). The last facet, Social Anxiety (SA), reflects the extent to which individuals focus on themselves when experiencing discomfort in front of others and is thought to be a consequence or result of Public SC (e.g., *I feel nervous when I speak in front of a group*) [31]–[33]. In the present study we used a French version validated by Pelletier & Vallerand (1990). The test-retest reliability was assessed with a 4-week interval between administrations. Test-retest correlations were .82 for Private subscale, .86 for Public subscale and .78 for Social Anxiety subscale indicating excellent stability across time [33].

### Statistical Analyses

All scales were administered to the patients at the beginning (T1 = day 1 or 2) and at the end (T2 = day 14 to 18) of the withdrawal treatment. Student's t values were used to compare variables from the self-report scales (obsession, compulsion, depression, Private SC, Public SC and Social Anxiety) between T1 and T2. Correlations between scores of depression, craving and SC questionnaires measured either at T1 or at T2 were calculated using Pearson-moment coefficients. We then tested whether SC moderate the relation between craving and depression. Hence, the relation between total craving scores and depression were calculated after splitting the population according to median scores at SC questionnaires. Furthermore, moderation analyses were conducted using a classical multiple linear regression model in which we examined the unique and interactive effects of depression state and trait SC on craving at both T1 and T2. In accord with Aiken and West's (1991) work, all continuous predictors (namely depression and SC) were centered around the mean [34]. In line with Judd and McClelland (1989), multivariate outliers deviating more than 3 standard deviations from the mean were removed in order to get the most accurate estimate of population parameters. In addition, to be as rigorous as possible in the subsequent incremental analyses (according to the principle of economy [35]) variables significantly or marginally significantly related to cravings were integrated in the multiple regressions. Finally, Bonferroni-corrections were applied to all statistical analyses.

## Results

Participants' ages ranged from 29 to 62 years with a mean of 47.7±8.8. All participants were French speaking. 63.3% were males. Forty-seven percent finished secondary school and 53% had a university degree. The number of years of addiction (i.e., since they lost control of their alcohol consumption) ranged from 1 to 34 years with a mean of 10.96±9.62. Moreover, 56.66% of these patients had at least one relative of their generation or the previous generation who had alcohol related problems. Forty percent of the patients had never entered a detoxification program before whereas 10% had made one attempt to quit drinking, 13% had made two attempts, 20% had made three attempts and 17% had made more than five attempts. The amount of alcohol consumed daily was 232.5±188 grams per day.

### Scores at the beginning (T1) and at the end (T2) of the protracted withdrawal for craving, depression and self-consciousness

#### Craving

**Figure 1:**
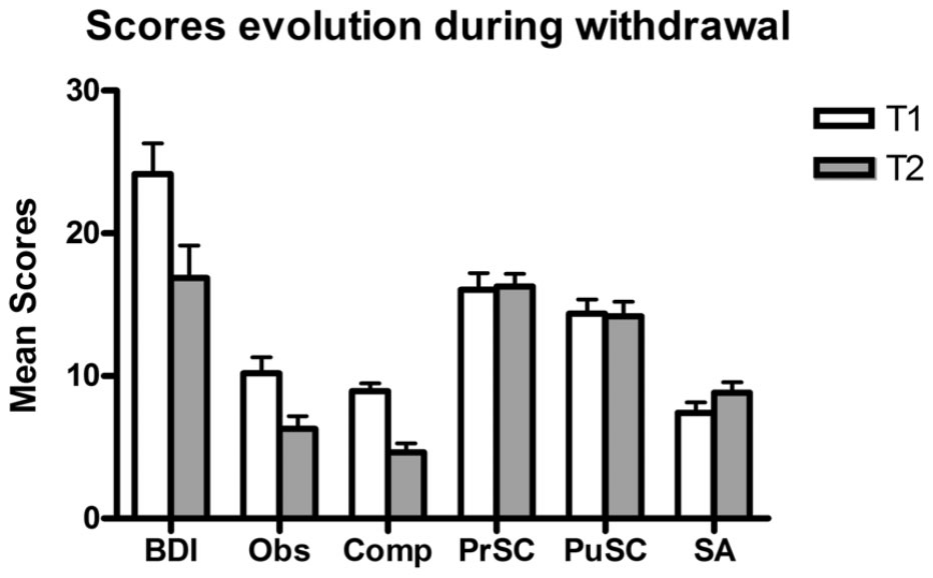
Evolution of scores of depression, craving and self consciousness during alcohol withdrawal. Depression scores were measured with the BDI (Beck et al., 1996; Bourque and Beaudette, 1982), obsessional (Obs) and compulsional (Comp) subscores of craving were measured by the OCDS (Anton et al., 1995, 1996; Ansseau et al., 2000), and self-consciousness were measured by the RSCS with subscores of Private (PrSC) and Public (PuSC) Self-Consciousness and Social Anxiety (SA) (Pelletier and Vallerand, 1990; Scheier and Carver, 1985) at both the onset (Time 1) and at the end (Time 2) of protracted withdrawal.

The internal consistencies of OCDS (obsessive and compulsive factors and total score) were good at both times (Cronbach's alpha for T1: Tot = .92; Ob = .92; Co = .84 and for T2: Tot = .93; Ob = .92; Co = .79) and were comparable to those of studies using both English and French validation questionnaires [25], [26]. Figure 1 shows that craving (Obsessive and compulsive scores) decreased significantly (*t* (29) = −4.498, p<0.0001) from the beginning to the end of withdrawal (*Cohen's ds* = .73).

#### Depression state

As shown in Figure 1, BDI-II scores decreased significantly from the beginning to the end of the withdrawal treatment (*t*(29) = −4.629, p<0.0001; *Cohen's ds* = .62). Additionally, the BDI-II Cronbach's alphas were identical at T1 and T2 (.93) and were comparable with those obtained in studies using both English [29] and French versions of the questionnaire [30].

#### Self-consciousness trait

Internal consistencies of the RSCS were acceptable and similar at both times (Cronbach's alpha for: Private SC = .82; Public SC = .89; SA = .70) and were comparable to studies using English and French studies validations of this scale [32], [33]. As shown in Figure 1, patients exhibited facets scores of SC that were stable (*t* (35) = 0.544, NS for global score).

### Correlation between craving and depressive state

To test the relation between craving and depressive state, we computed Pearson-moment correlations coefficients (r) between OCDS (obsessive factor, compulsive factor and total score) and BDI scores. Our results indicated significant positive correlations between both sub-factors of craving (obsession and compulsion) and depression scores at both times (see Table 1). Moreover, the longitudinal correlations (i.e., involving BDI at T1 and OCDS at T2) were still significant (i.e., with the same level of significance) when OCDS T1 was included in the analysis as a covariate variable (i.e., partial correlation by controlling for OCDS T1).

**Table 1:** Correlation matrix between variables at T1&T2 with each variables overall Mean and SD at T1&T2.

	BDIT1	ObT1	CoT1	cravingT1	PrSCT1	PuSCT1	SAT1	SCT1	BDIT2	ObT2	CoT2	cravingT2	PrSCT2	PuSCT2	SAT2	SCT2	Mean	SD
**BDIT1**	-	.58***	.48**	.57***	−.24	−.15	.30	−.09	.72***	.60***	.47**	.58***	−.20	−.08	−.02	−.13	24.17	11.75
**ObT1**		-	.72***	.93***	.06	−.19	.18	.01	.33†	.31†	.24	.30	−.07	−.23	−.18	−.21	10.20	6.15
**CoT1**			-	.93***	−.01	−.18	.18	−.02	.08	.15	.07	.13	.02	−31	−.20	−.21	8.93	3.09
**TotT1**				-	.02	−.20	.19	−.01	.22	.25	.17	.23	−.02	−.29	−.21	−.23	19.13	8.40
**PrSCT1**					-	.69***	.38*	.92***	−.35†	−.28	−.30	−.30	.62***	.50***	.22	.61***	16.07	6.22
**PuSCT1**						-	.12	.81***	−.21	−.25	−.30	−.30	.42**	.54***	.07	.48***	14.40	5.26
**SAT1**							-	.59***	.15	.02	−.06	−.01	.17	−.03	.18	.12	7.43	3.96
**SCT1**								-	−.21	−.24	−.31	−.28	.54***	.46**	.20	.55***	37.90	12.56
**BDIT2**									-	.60***	.47***	.57***	−.26	.02	−.01	.10	16.87	12.44
**ObT2**										-	.82***	.97***	−.16	−.16	−.10	−.18	6.30	4.88
**CoT2**											-	.93***	−.40*	−.23	−.01	−.29	4.67	3.39
**TotT2**												-	−.27	−.20	−.07	−.23	10.97	8.23
**PrSCT2**													-	.58***	.36*	.86***	16.30	4.73
**PuSCT2**														-	.12	.80***	14.20	5.53
**SAT2**															-	.59***	8.83	4.03
**SCT2**																-	39.33	11.22

† .05<*p*<.10,

* p<.05,

** p<.01,

*** p<.001.

Abbreviations: T1 = Time 1, T2 = Time 2, BDI = Beck Depression Inventory, Ob = Obsessive factor, Co = Compulsive factor, Tot = Total craving, PrSC = Private Self-Consciousness; PuSC = Public Self-Consciousness; SA = Social Anxiety, SC = Self-Consciousness.

Values in the tables represent the Pearson-moment correlation coefficient (r).

Values in the tables represent the Pearson-moment correlation coefficient (r).

### Correlation between SC and depressive state or craving

No significant Pearson-moment correlations between SC and BDI scores or between SC and OCDS scores emerged in our analyses (see Table 1).

### Moderating effect of trait SC on the relation between depression and craving

Before studying the moderating effect of SC on the association between depression and craving, multiple regressions were computed with socio-demographic variables (age and years of addiction) as predictors and the total craving score and its two subscales as dependent variables. We conducted this analysis in order to test whether both socio-demographic factors influenced our craving scores. Our results showed no impact of these socio-demographic factors on craving for alcohol (obsessive and compulsive factors, at T1 and T2) (adjusted R^2^ less than .07). Age of patients was the only predictor of obsession at T2 (adjusted R^2^ = .19). For this reason, we took into account age in the following analyses.

**Figure 2:**
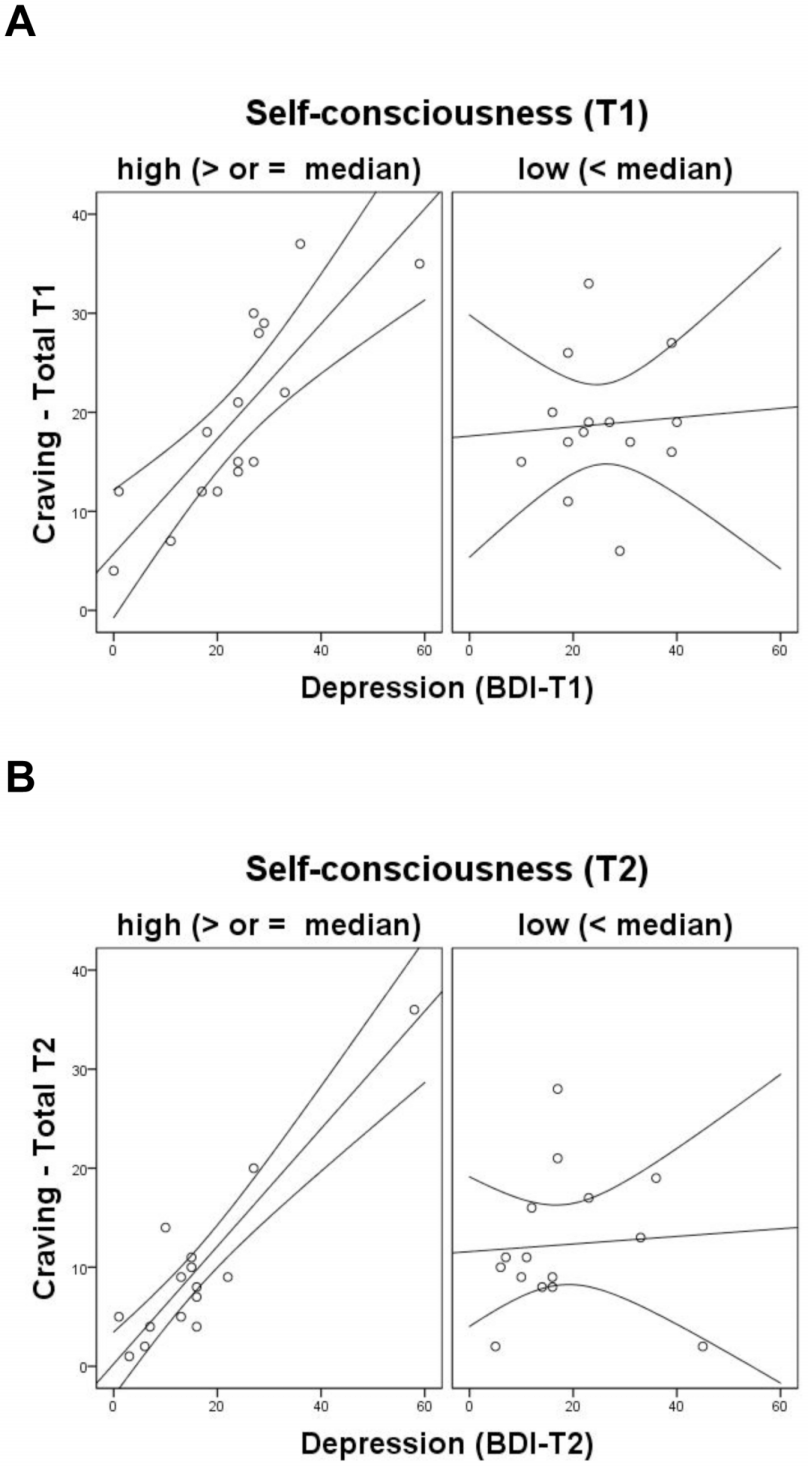
Relation between craving and depression scores as a function of self-consciousness. The relations were depicted for T1 (panel A) and T2 (panel B). In the four graphs, the curves around the regression line delineate a 95% confidence relation between craving and depression scores.

We examined whether SC moderates the association between depressive state scores and craving scores at the beginning and at the end of the withdrawal treatment. As shown in Tables 2 and 3, our analyses revealed interactions between depression (BDI) and only two SC dimensions (Private SC and Public SC) and the total SC was a significant predictors of craving for alcohol for both the OCDS global score and two subscales (obsessive and compulsive factors). We found no significant interaction between BDI and SA. We did, however, find a significant interaction between BDI and Private SC which suggests that the positive relation between depression and craving was stronger when patients had higher Private SC scores. We also found an interaction between BDI and Public SC, on the one hand, and BDI and Total SC, on the other hand. It is important to note, however, that the interactions at T1 were only marginal while the interactions at T2 were significant. Overall, these results suggested that for low SC (i.e., Public SC and Private SC) patients, their depression levels did not affect their craving scores both at the beginning and at the end of the withdrawal treatment. However, for high SC (i.e., Public SC and Private SC) patients, depression scores were positively related to craving (i.e., obsessive and compulsive factors) (See Figures 2A and 2B).

**Table 2:** Multiple regression analyses predicting craving scores (T1) as a function of depression T1, SC T1 and their interaction.

			Craving (OCDS)			
	Obsessive factor		Compulsive factor		Total score	
	Bêta	t	Bêta	t	Bêta	t
**Private SC**	R^2^adj = .43		R^2^adj = .33		R^2^adj = .45	
	F (3,26) = 5.46*		F (3,26) = 6.80**		F (3, 26) = 8.08**	
BDI T1	.51	3.29**	.36	2.13*	.46	3.08**
Private SC T1	.20	1.39	.10	.62	.16	1.13
Interaction	.35	2.34*	.42	2.61**	.42	2.84**
**Public SC**	R^2^adj = .30		R^2^adj = .26		R^2^adj = .33	
	F (3, 26) = 2.08†		F (3, 26) = 2.89†		F (3, 26) = 2.70†	
BDI T1	.49	2.90**	.33	1.89†	.45	2.67**
Public SC T1	−.04	−.25	.02	.92	−.01	−.08
Interaction	.29	2.04†	.26	1.97†	.30	1.64†
**Social anxiety**	R^2^adj = .32		R^2^adj = .15		R^2^adj = .30	
	F (3, 26) = 1.78		F (3, 26) = .04		F (3, 26) = 1.49	
BDI T1	.58	3.83***	.47	2.62*	.57	3.48**
Social anxiety T1	.01	.05	.03	.19	.02	.14
Interaction	.20	1.26	.04	.21	.19	1.22
**Total SC**	R^2^adj = .46		R^2^adj = .24		R^2^adj = .44	
	F (3, 26) = 5.63*		F (3, 26) = 3.18†		F (3, 26) = 7.27**	
BDI T1	.42	2.84**	.34	1.90†	.42	2.85**
Total SC T1	.07	.14	.03	.92	.11	.72
Interaction	.38	2.61*	.32	1.96†	.45	2.97**

* *p*<0.05,

** *p*<0.01,

*** *p*<0.001,

† *p*<0.1.

**Table 3:** Multiple regression analyses predicting craving scores (T2) as a function of depression T2, SC T2, and their interaction.

			Craving (OCDS)			
	Obsessive factor		Compulsive factor		Total score	
	Bêta	t	Bêta	t	Bêta	t
**Private SC**	R^2^adj = .64		R^2^adj = .43		R^2^adj = .54	
	F (4,25) = 20.77***		F (3,26) = 9.04**		F (3, 26) = 15.23***	
Age	.29	2.50*	-	-	-	-
BDI T2	.51	4.23***	.36	2.51*	.50	3.84***
Private SC T2	−.02	−.19	−.36	−2.47*	−.21	−1.56
Interaction	.52	4.55***	.43	3.01**	.50	3.90***
**Public SC**	R^2^adj = .59		R^2^adj = .34		R^2^adj = .52	
	F (4, 25) = 15.39***		F (3, 26) = 5.64*		F (3, 26) = 11.73***	
Age	.23	1.85†	-	-	-	-
BDI T2	.39	3.03**	.36	2.29*	.43	3.18**
Public SC T2	−.06	−.47	−.02	−1.33	−.16	−1.22
Interaction	.49	3.92***	.38	2.37*	.47	1.42**
**Social anxiety**	R^2^adj = .35		R^2^adj = .19		R^2^adj = .28	
	F (4, 25) = .32		F (3, 26) = 1.78		F (3, 26) = .85	
Age	.27	1.73†	-	-	-	-
BDI T2	.56	3.41**	.55	3.11**	.62	3.71***
Social anxiety T2	.19	−.41	.04	.24	−.03	−.19
Interaction	.02	.56	.24	1.33	.16	.92
**Total SC**	R^2^adj = .62		R^2^adj = .36		R^2^adj = .52	
	F (4, 25) = 18.43***		F (3, 26) = 6.53*		F (3, 26) = 12.84***	
Age	.28	2.29*		-		-
BDI T2	.48	4.06***	.42	2.80**	.52	3.97***
Total SC T2	.03	.24	−.18	−1.16	−.10	−.74
Interaction	.50	4.29***	.39	2.55*	.47	3.58***

* *p*<0.05,

** *p*<0.01,

*** *p*<0.001,

† *p*<0.1.

Finally, a similar analysis was conducted on the longitudinal relation between depressive symptoms at T1 and craving at T2 with T1 craving scores as a covariate. This analysis, however, revealed no significant interactions between BDI and SC (Private SC, Public SC, SA) on OCDS (obsession, compulsion and total score).

## Discussion

Our first aim of this study was to investigate the evolution of depression state and self-reported craving for alcohol and their relation during a withdrawal and detoxification program. A large decrease of depressive symptoms was observed from T1 to T2. That is, when the patients started, they presented a moderate level of depression while, at end of the withdrawal treatment, they only exhibited mild depression levels.

Moreover, this large decrease of depression observed among alcohol-dependent patients during the 3-week protracted withdrawal and detoxification program was mirrored by a decrease in self-reported craving (for both obsessive and compulsive factors), a result often found in previous studies [6], [8]. Additionally, significant correlations between depression state and self-reported craving occurred at both times of the treatment, again, replicating previous research [6], [9]. The longitudinal relation between depression state at the beginning and craving state at the end of the treatment has sometimes been interpreted as a causal relation [9]. This causal interpretation was supported by the fact that the relation between depression and craving were still observed after controlling for craving scores at the beginning of the treatment (the baseline covariate). Furthermore, given the frequent disordered self-referential thoughts (i.e. ruminations) characteristic of depression [36], these results suggest that abnormal self-referential processes associated with depression may increase craving for alcohol. Therefore, additional research is needed to examine the relations depression and craving. The role of the default mode network, a group of cerebral areas that exert in human a function for self-referential processes should be tested [37] as well as that of the neural circuits involved in self-awareness (i.e., insula and medial regions of prefrontal cortex) which are often found to be defective in addicts.

Our second aim was to examine whether trait SC moderates the association between depressive state and self-reported craving. First, SC scores observed among alcohol-dependent patients, both at T1 and T2, are similar to scores of healthy French-speaking subjects [33]. The absence of change of SC scores from the beginning to the end of protracted withdrawal is not surprising since the scores do not differ from the general population and since the scale measures a stable trait. Second, our results indicate that patients with high levels of depressive symptoms are likely to suffer from craving both at the beginning and at the end of a 3-week withdrawal program if they have a high SC score. In other words, the higher the SC score, the greater the influence of depressive state on craving. This moderation of trait SC was due to Private SC and Public SC subscales, but not to the Social Anxiety subscale.

These results were consistent with the SC model of alcohol consumption proposed by Hull [19]. Hull's model suggests that alcohol decreases an individuals' level of SC. Our results support this assumption, as alcohol-dependent patients, who exhibit high SC scores, show a strong craving when exhibiting depressive symptoms at both the beginning and more importantly at the end of the detoxification program. Our results suggest that alcohol might act as an “antidepressant” leading to a decrease of self-related thoughts. In addition, high levels of SC may be a possible factor of relapse after a 3-week withdrawal treatment when the patients are exposed to negative events or thoughts, as observed by Hull and colleagues [17]. Additionally, some studies investigating longitudinal treatment effects of alcohol-dependence demonstrated a positive correlation between craving intensity and relapse severity [10]–[12]. Therefore, our findings provide evidence that craving might be an intermediate process with respect to the way Hull observed a moderation of SC scores on the relation between experiences of failure and the risk of relapse. Consequently, it is critical for future researchers to systematically control for depression and SC when patients start a detoxification program. Among such patients, those with high depression and high SC would be a specific group to target for risks for relapses, whereas the high depressed but low SC patients would not.

Moreover, the negative aspects of Private SC and Public SC may be related to self-stigma and shame (i.e., social stigma). These two affective dimensions have often been considered as barriers to alcohol treatment [38]. Indeed, Private SC is related to an individual's own negative self-view or self-stigma [39] which has been shown to lower self–esteem [40]. Indeed, self-stigma would lead to pursuit of alcohol consumption when the individual cannot find another way to reduce or eliminate these negative self-feelings.

Conversely, shame is a negative reaction to others' views and opinions and is related to Public SC. Addiction is among various psychiatric disorders the one which induces the strongest tendency to be stigmatized by others [41], even caregivers [42]. Furthermore, addicts are very sensitive to ostracism [41]. Recently, social stigma (i.e., shame) has been found to be associated with increased depressiveness, anxiety and poor quality of life in drug addiction, leading to a vicious circle [43], [44]).

Clinically speaking, our results suggest that clinicians should focus on reducing the strong association between depressive symptoms and craving at both times of the treatment process [45]. Moreover, our results indicate that some individual differences critically shape this association. Therefore, clinicians should focus on patients exhibiting high SC scores. Although SC scores did not change during this 3-week protracted withdrawal and detoxification program, this does not mean that it may not evolve after withdrawal or that some interventions cannot modify their sensibility to self-stressors (i.e. disordered self-related thoughts) during or after withdrawal. Indeed, high SC patients might benefit from specific interventions to modify this sensibility. Metacognitive therapy such as mindfulness-based strategies may help alcohol-dependent patients. This kind of therapy emphasizes acceptance strategies, non-judgment and non-reaction to thoughts, feelings and sensations. Recently, a mindfulness-based relapse prevention treatment was used in adults with substance abuse disorders. At the end of the treatment, these patients exhibited a decrease in craving and increases in acceptance as compared to a control group [46]. Researchers have also found such treatments to decrease depression and alcohol craving scores [47], [48].

Our study, however, does have a few minor limitations. First, our sample size is relatively small. Despite this, we still find medium to strong effect sizes (For mean comparison tests: Cohen's *d*s>.60 and for moderation analyses: Rs^2^ Adj>.24) suggesting that our results are reliable. Second, subjects were administered some medication (i.e., benzodiazepines) during detoxification treatment which may have confounded our results. Indeed, medication effects cannot be teased apart from the detoxification effects in our results. Nevertheless, the dosages of benzodiazepines were entered into the analyses as a covariate, but no effect of this medication emerged. In any event, this confound is an unavoidable byproduct of conducting this type of research according to strict ethical guidelines. Furthermore, it is highly unethical to prevent patients from being given any medication during a detoxification program. Lastly, our craving measure was based on self-reporting, with no corroborating objective measures of craving (e.g. heteroanamnesia, biological parameters analysis, cue-reactivity to alcohol stimuli or neuroimagery [7], [49]). Although these techniques can be applied and used in a clinical setting, they are expensive and not always available, as in our current study.

Future research should focus on further examining the role of individual differences in better understanding the associations between depression and the various components of alcoholism (i.e., craving, withdrawal, and relapse). For example, some researchers [50], [51] have shown how neuroticism and/or emotional intelligence moderates the association between affect and alcohol use. Such research will improve not only alcohol treatments in general, but also the procedure through which treatments are chosen for particular patients.

## References

[1] Schuckit MA (1994) Alcohol and depression: a clinical perspective. Acta Psychiatrica Scandinavica 89: 28–32. 10.1111/j.1600-0447.1994.tb05798.x 8053363

[2] Schuckit MA, Tipp JE, Bergman M, Reich W, Hesselbrock VM, et al (1994) Comparison of induced and independent major depressive disorders in 2945 alcoholics. The American Journal of Psychiatry 154: 948–957. 10.1176/ajp.154.7.948 9210745

[3] Haviland MG, MacMurray JP, Cummings MA (1988a) The relationship between alexithymia and depressive symptoms in a sample of newly abstinent alcoholic inpatients. Psychotherapy and Psychosomatics 49: 37–40. 3237960 10.1159/000288065

[4] Pinard L, Negrete JC, Annable L, Audet N (1996) Alexithymia in substance abusers-persistence and correlates of variance. The American Journal on Addictions 5: 32–39.

[5] de Timary P, Luts A, Hers D, Luminet O (2008) Absolute and relative stability of alexithymia in alcoholic inpatients undergoing alcohol withdrawal: Relationship to depression and anxiety. Psychiatry Research 157: 105–113. 17884180 10.1016/j.psychres.2006.12.008

[6] Andersohn F, Kiefer F (2004) Depressive mood and craving during alcohol withdrawal: Association and interaction. The German Journal of Psychiatry 7: 6–11.

[7] Anton RF (1999) What is craving. Alcohol Research and Health 23: 165–173. 10890811 PMC6760371

[8] Cordovil de Sousa Uva M, Luminet O, Cortesi M, Constant E, Derély M, et al (2010b) Distinct effects of protracted withdrawal on affect, craving, selective attention and executive functions among alcohol-dependent patients. Alcohol & Alcoholism 45: 241–246. 20207627 10.1093/alcalc/agq012

[9] Cordovil de Sousa Uva M, de Timary P, Cortesi M, Mikolajczak M, du Roy de Blicquy P, et al (2010a) Moderating effect of emotional intelligence on the role of negative affect in the motivation to drink in alcohol-dependent subjects undergoing protracted withdrawal. Personality and Individual Differences 48: 16–21.

[10] Paille F, Guelfi JD, Perkins AC, Royer RJ, Steru L, et al (1995) Double-blind randomized multicentre trial of acamprosate in maintaining abstinence from alcohol. Alcohol & Alcoholism 30: 239–247. 7662044

[11] Volpicelli JR, Alterman AI, Hayashida M, O'Brien CP (1992) Naltrexone in the treatment of alcohol dependence. Archives of General Psychiatry 49: 876–880. 1345133 10.1001/archpsyc.1992.01820110040006

[12] O'Malley SS, Jaffe AJ, Chang AJ, Schottenfeld RS, Meyer RE, et al (1992) Naltrexone and coping skills therapy for alcohol dependence. A controlled study. Archives of General Psychiatry 49: 881–887. 1444726 10.1001/archpsyc.1992.01820110045007

[13] Baker TB, Piper ME, McCarthy DE, Majeskie MR, Fiore MC (2004) Addiction motivation reformulated: An affective processing model of negative reinforcement. Psychological Review 111: 33–51. 14756584 10.1037/0033-295X.111.1.33

[14] Brandon TH, Wetter DW, Baker TB (1996) Affect, Expectancies, Urges, and smoking : Do they conform to models of drug motivation and relapse. Experimental and Clinical Psychopharmacology 4: 29–36.

[15] Oslin DW, Cary M, Slaymaker V, Colleran C, Blow FC (2009) Daily ratings measures of alcohol craving during an inpatient stay define subtypes of alcohol addiction that predict subsequent risk for resumption of drinking. Drug and Alcohol Dependence 103: 131–136. 19443131 10.1016/j.drugalcdep.2009.03.009 PMC12272362

[16] Littlefield AK, Sher KJ (2010) The multiple distinct ways that personality contributes to alcohol use disorders. Social and Personality Psychology Compass 4: 767–782. 21170162 10.1111/j.1751-9004.2010.00296.x PMC3002230

[17] Hull JG, Young RD, Jouriles E (1986) Applications of the self-awareness model of alcohol consumption: Predicting patterns of use and abuse. Journal of Personality and Social Psychology 51: 790–796. 3783425 10.1037//0022-3514.51.4.790

[18] Maurage P, de Timary P, Moulds ML, Wong QJ, Collignon M, et al (2013) Maladaptive Social Self-Beliefs in Alcohol-Dependence: A Specific Bias towards Excessive High Standards. PLoS One 8: e58928. 23520543 10.1371/journal.pone.0058928 PMC3592810

[19] Hull JG (1981) A self-awareness model of the causes and effects of alcohol consumption. Journal of Abnormal Psychology 90: 586–600. 7320328 10.1037//0021-843x.90.6.586

[20] DiClemente CCP, James O (1998) Toward a comprehensive, transtheoretical model of change: Stages of change and addictive behaviors. ; Miller WRH, Nick editor. New YorkNYUS: Plenum Press. 357 p.

[21] Hull JG, Reilly NP (1983) Self-awareness, self-regulation, and alcohol consumption: a reply to Wilson. J Abnorm Psychol 92: 514–519. 6643830

[22] APA (1994) Diagnostic and statistical manual of mental disorders (DSM -IV). Washington, DC.

[23] Kosten TR, O'Connor PG (2003) Management of drug and alcohol withdrawal. The New England Journal of Medecine 348: 1786–1795. 10.1056/NEJMra020617 12724485

[24] Anton RF, Moak DH, Latham PK (1995) The Obsessive Compulsive Drinking Scale: A self-rated instrument for the quantification of thought about alcohol and drinking behaviour. Alcoholism: Clinical and Experimental Research 19: 92–99. 10.1111/j.1530-0277.1995.tb01475.x 7771669

[25] Anton RF, Moak DH, Latham PK (1996) The Obsessive Compulsive Drinking Scale: A new method of assessing outcome in alcoholism treatment studies. Archives of General Psychiatry 53: 225–231. 8611059 10.1001/archpsyc.1996.01830030047008

[26] Ansseau M, Besson J, Lejoyeux M, Pinto E, Landry U, et al (2000) A French translation of the Obsessive-Compulsive Drinking Scale for craving in alcohol-dependent patients: a validation study in Belgium, France, and Switzerland. European Addiction Research 5: 51–56. 10.1159/000019010 10899729

[27] Clark DC, Gibbons RD, Fawcett J, Aagesen CA, Sellers D (1985) Unbiased criteria for severity of depression in alcoholic inpatients. Journal of Nervous and Mental Disorders 173: 482–487. 10.1097/00005053-198508000-00005 4020366

[28] Clark DC, Gibbons RD, Haviland MG, Hendryx MS (1993) Assessing the severity of depressive states in recently detoxified alcoholics. Journal of Studies on Alcoholism 54: 107–114. 10.15288/jsa.1993.54.107 8355494

[29] Beck AT, Steer RA, Brown J (1996) Manual for the Beck Depression Inventory-II. San Antonio, TX.

[30] Bourque P, Beaudette D (1982) Etude psychométrique du questionnaire de dépression de Beck auprès d'un échantillon d'étudiants universitaires francophones. Revue canadienne des sciences du comportement 14: 211–218.

[31] Scheier MF, Carver CS (1985) The self-consciousness scale: a revised version for use with general population. Journal of Applied Social Psychology 15: 687–699.

[32] Fenigstein A, Scheier MF, Buss AH (1975) Public and private self-Consciousness : assessment and theory. Journal of Consulting and Clinical Psychology 43: 522–527.

[33] Pelletier LG, Vallerand RJ (1990) L'Echelle Révisée de Conscience de Soi: une traduction et une validation canadienne-française du Revised Self-Consciousness Scale. Revue Canadienne des Sciences du Comportement 22: 191–206.

[34] Aiken LS, West SG (1991) Multiple regression: Testing and interpreting interactions. Thousand Oaks: Sage Publications.

[35] Judd CM (1989) Data analysis: A model-comparison approach. San diego: Harcourt Brace Jovanovich.

[36] Sheline YI, Barch DM, Price JL, Rundle MM, Vaishnavi SN, et al (2009) The default mode network and self-referential processes in depression. Proceedings of the National Academy of Sciences of the United States of America 106: 1942–1947. 19171889 10.1073/pnas.0812686106 PMC2631078

[37] Buckner RL, Andrews-Hanna JR, Schacter DL (2008) The Brain's Default Network. Annals of the New York Academy of Sciences 1124: 1–38. 18400922 10.1196/annals.1440.011

[38] Saunders SM, Zygowicz KM, D'Angelo BR (2006) Person-related and treatment related barriers to alcohol treatment. Journal of Substance Abuse Treatment 30: 261–270. 16616171 10.1016/j.jsat.2006.01.003

[39] Corrigan P (2004) How stigma interferes with mental health care. The American psychologist 59: 614–625. 15491256 10.1037/0003-066X.59.7.614

[40] Wahl OF (1999) Mental health consumers' experience of stigma. Schizophrenia Bulletin 25: 467–478. 10478782 10.1093/oxfordjournals.schbul.a033394

[41] Schomerus G, Lucht M, Holzinger A, Matschinger H, Carta MG, et al (2011) The stigma of alcohol dependence compared with other mental disorders: a review of population studies. Alcohol Alcohol 46: 105–112. 21169612 10.1093/alcalc/agq089

[42] Luoma JB, Twohig MP, Waltz T, Hayes SC, Roget N, et al (2007) An investigation of stigma in individuals receiving treatment for substance abuse. Addict Behav 32: 1331–1346. 17092656 10.1016/j.addbeh.2006.09.008

[43] Frischknecht U, Beckmann B, Heinrich M, Kniest A, Nakovics H, et al (2011) The vicious circle of perceived stigmatization, depressiveness, anxiety, and low quality of life in substituted heroin addicts. Eur Addict Res 17: 241–249. 21654177 10.1159/000328637

[44] Toussaint A, de Timary P (2006) [Drawing the alcoholic out of his isolation]. J Pharm Belg 61: 37–44. 16669346

[45] Witkiewitz K, Bowen S (2010) Depression, craving, and substance use following a randomized trial of mindfulness-based relapse prevention. J Consult Clin Psychol 78: 362–374. 20515211 10.1037/a0019172 PMC3280693

[46] Bowen S, Chawla N, Collins SE, Witkiewitz K, Hsu S, et al (2009) Mindfulness-based relapse prevention for substance use disorders: a pilot efficacy trial. Substance Abuse 30: 294–305. 10.1080/08897070903250084 PMC3280682 19904665

[47] Deyo M, Wilson KA, Ong J, Koopman C (2009) Mindfulness and Rumination: Does Mindfulness Training Lead to Reductions in the Ruminative Thinking Associated With Depression? The Journal of Science and Healing 5: 265–271. 10.1016/j.explore.2009.06.005 19733812

[48] Witkiewitz K, Bowen S (2010) Depression, craving, and substance use following a randomized trial of mindfulness-based relapse prevention. Journal of Consulting and Clinical Psychology 78: 362–374. 20515211 10.1037/a0019172 PMC3280693

[49] Weinstein A, Lingford-Hughes A, Martinez-Raga J, Marshall J (1998) What makes alcohol-dependent individuals early in abstinence crave for alcohol: exposure to the drink, images of drinking, or remembrance of drinks past? Alcohol Clin Exp Res 22: 1376–1381. 9756056

[50] Armeli S, Tennen H, Todd M, Carney MA, Mohr C, et al (2003) A daily process examination of the stress-response dampening effects of alcohol consumption. Psychology of addictive behaviors 17: 266–276. 14640822 10.1037/0893-164X.17.4.266

[51] Carney MA, Armeli S, Tennen H, Affleck G, O'Neil TP (2000) Positive and negative daily events, perceived stress and alcohol use: A diary study. Journal of Counseling and Clinical Psychology 68: 788–798. 11068965

